# Evaluating the effect of R-Baclofen and LP-211 on autistic behavior of the BTBR and *Fmr1*-KO mouse models

**DOI:** 10.3389/fnins.2023.1087788

**Published:** 2023-03-30

**Authors:** Shirin Sharghi, Stefanie Flunkert, Magdalena Daurer, Roland Rabl, Boris Philippe Chagnaud, Marcello Leopoldo, Enza Lacivita, Birgit Hutter-Paier, Manuela Prokesch

**Affiliations:** ^1^Department of Neuropharmacology, QPS Austria GmbH, Grambach, Austria; ^2^Institute for Biology, Karl-Franzens-Universität Graz, Graz, Austria; ^3^Department of Pharmacy-Drug Sciences, University of Bari Aldo Moro, Bari, Italy

**Keywords:** autism spectrum disorders, locomotor behavior, anxiety, repetitive behavior, ultrasonic vocalization, GABA_*B*_ receptor agonist, 5-HT_7_ receptor agonist

## Abstract

**Introduction:**

Autism spectrum disorder (ASD) is a persistent neurodevelopmental condition characterized by two core behavioral symptoms: impaired social communication and interaction, as well as stereotypic, repetitive behavior. No distinct cause of ASD is known so far; however, excitatory/inhibitory imbalance and a disturbed serotoninergic transmission have been identified as prominent candidates responsible for ASD etiology.

**Methods:**

The GABA_*B*_ receptor agonist R-Baclofen and the selective agonist for the 5HT_7_ serotonin receptor LP-211 have been reported to correct social deficits and repetitive behaviors in mouse models of ASD. To evaluate the efficacy of these compounds in more details, we treated BTBR *T^+^ Itpr3*^tf^*/J* and B6.129P2-*Fmr1*^*tm*1*Cgr*^/*J* mice acutely with R-Baclofen or LP-211 and evaluated the behavior of animals in a series of tests.

**Results:**

BTBR mice showed motor deficits, elevated anxiety, and highly repetitive behavior of self-grooming. *Fmr1*-KO mice exhibited decreased anxiety and hyperactivity. Additionally, *Fmr1*-KO mice’s ultrasonic vocalizations were impaired suggesting a reduced social interest and communication of this strain. Acute LP-211 administration did not affect the behavioral abnormalities observed in BTBR mice but improved repetitive behavior in *Fmr1*-KO mice and showed a trend to change anxiety of this strain. Acute R-Baclofen treatment improved repetitive behavior only in *Fmr1*-KO mice.

**Conclusion:**

Our results add value to the current available data on these mouse models and the respective compounds. Yet, additional studies are needed to further test R-Baclofen and LP-211 as potential treatments for ASD therapy.

## 1. Introduction

Autism spectrum disorder (ASD) is a group of complex lifelong neurodevelopmental conditions affecting around 1−1.5% of the global population (Bougeard et al., 2021; Zeidan et al., 2022). The male-female incidence of ASD is 4:1 to 3:1 which has been repetitively observed and the focus of the ASD research lies therefore on the male population (Loomes et al., 2017; Posserud et al., 2021). Hallmark ASD symptoms are categorized as unusual reciprocal social communication and impaired communication as well as stereotypic, repetitive behaviors with restricted interest. Seizure, anxiety, and hyperactivity are comorbid ASD symptoms with an incidence of 40−78% (Murray, 2010; Zaboski and Storch, 2018). One important hypothesis consistent with these comorbidities is an imbalance of excitatory/inhibitory (E/I) neurotransmission in ASD (Geschwind and Levitt, 2007; Bourgeron, 2009; Gogolla et al., 2014; Uzunova et al., 2016; Culotta and Penzes, 2020; Canitano and Palumbi, 2021). Reduced GABAergic neurotransmission and fewer GABAergic interneurons are summarized as reduced GABAergic action in the autistic brain that has been detected both in humans and animal models (Kubas et al., 2012; Han et al., 2014; Cochran et al., 2015; Silverman et al., 2015; Robertson et al., 2016; Möhrle et al., 2021). Furthermore, genetic evidence strongly supports that the E/I imbalance effects ASD pathogenesis (Delahanty et al., 2011; Bonnet-Brilhault et al., 2016; Guo et al., 2017; Noroozi et al., 2018).

Hyperserotonemia is the first and most replicated biochemical abnormality identified in autistic individuals (Schain and Freedman, 1961; Anderson et al., 1987; Folk and Long, 1988; Leboyer et al., 1999; Pagan et al., 2014; Muller et al., 2016). The disturbed serotoninergic (5-hydroxytryptamine, 5-HT) system was identified as an underlying cause of neuropsychiatric disorders such as ASD, and manipulation of 5-HT signaling was consequentially shown to improve ASD-like behaviors in animal models (Kolevzon et al., 2006; Leopoldo et al., 2011; Costa et al., 2012, 2018; Lacivita et al., 2017; Saitow et al., 2020; Andersson et al., 2021; Lee et al., 2021).

BTBR *T*^+^ *Itpr3^tf^*/J mice (BTBR) are a widely used inbred mouse model that robustly exhibits ASD phenotypes of highly repetitive behaviors (Silverman et al., 2010a,^2015^), anxiety, exaggerated stress response (Benno et al., 2009; Pobbe et al., 2011), as well as social impairments (Silverman et al., 2015; Amodeo et al., 2021). Social approach impairment in BTBR mice can be improved by the application of the GABA_*A*_R-active neurosteroid ganaxolone (Kazdoba et al., 2016). Another GABAergic compound, clonazepam, improved sociability in BTBR mice (Han et al., 2014). The selective GABA_*B*_ receptor agonist, R-Baclofen, is reported to reverse social deficits and repetitive behavior in BTBR mice (Silverman et al., 2015). This mouse model is shown to have reduced serotonin transporter (SERT) activity in various brain regions as well as increased 5-HT_1*A*_ receptor levels in the hippocampus (Speranza et al., 2017).

Another model to investigate ASD is the Fragile X syndrome (FXS) mouse model. FXS is the most frequent form of inherited intellectual disability and monogenic cause of ASD affecting 1/4,000 male and 1/7,000 female subjects (Rogers et al., 2001; Kazdoba et al., 2014). FXS is caused by a CGG repeat mutation on the X chromosome containing the *FMR1* gene resulting in hypermethylation and therefore silencing of this gene leading to reduced levels of the Fragile X mental retardation protein (FMR1P) (Telias, 2019). *Fmr1*-knock out (KO) mice present hyperactivity, repetitive behavior, seizure, and social impairments as well as altered anxiety, thus recapitulating FXS symptoms of humans (Bakker et al., 1994; Spencer et al., 2005; Mineur et al., 2006; Olmos-Serrano et al., 2011; Pietropaolo et al., 2011; Rotschafer et al., 2012; Dolan et al., 2013; Qin et al., 2015). Increased dendritic spine density and abnormal spinal morphology are known neuronal features in *Fmr1*-KO mice (Comery et al., 1997; Booker et al., 2019). These mice also present alterations in Group I metabotropic glutamate receptor type 5 (mGlu5) and GABAergic system deficits in the cortex, hippocampus, amygdala, striatum, and subiculum (Paluszkiewicz et al., 2011; Booker et al., 2019; Afshar et al., 2022). Decreased expression of GABAergic signaling combined with an increase in glutamatergic pathway activity results in hyperexcitability of these mice (Gibson et al., 2008; Paluszkiewicz et al., 2011).

Chemicals correcting this hyperexcitation could be useful as potential therapeutic to treat FXS-related symptoms. R-Baclofen (STX209), the most potent enantiomer of Baclofen (Lal et al., 2009), has already been used to alleviate FXS behaviors by an acute treatment as well as to correct spine density by a chronic treatment (Henderson et al., 2012). Moreover, the acute R-Baclofen treatment was shown to significantly improve auditory-evoked gamma oscillations, but not the impaired sociability in *Fmr1*-KO mice (Sinclair et al., 2017).

Based on results from preclinical studies, clinical studies on R-Baclofen were performed, resulting in some side effects such as sedation of the animals, but it was concluded that R-Baclofen is a potential candidate to improve sociability in FXS patients (Berry-Kravis et al., 2012). A phase 3 clinical study with R-Baclofen that was based on caregivers’ questionnaires, however, reported a lack of efficacy in FXS patients (Berry-Kravis et al., 2017). No further clinical studies reported the effect of R-Baclofen in FXS patients since then.

Moreover, FXS-related behaviors in mice are improved by serotonergic compounds. One such compound is LP-211 that reverses mGluR mediated long-term depression (LTD) in the hippocampus of wild type and *Fmr1*-KO mice and improves learning deficits as well as repetitive behavior in these mice (Costa et al., 2012, 2015). Recent work from Costa et al. (2021) further confirmed the LP-211 effect on mGluR-LTD and revealed that this compound requires cyclin-dependent kinase 5 to rescue abnormal plasticity in *Fmr1*-KO mice.

In the present study, we characterize BTBR and *Fmr1*-KO mice for their ASD-related behaviors by assessing locomotor activity, anxiety-like, and repetitive behaviors. In addition, the communication of *Fmr1*-KO mice is tested using the ultrasonic vocalization test. Furthermore, we evaluate the efficacy of the highly discussed GABAergic agonist R-Baclofen and the serotonergic compound LP-211 in both mouse models to explore whether treatment with these compounds can rescue ASD-associated behavioral phenotypes.

## 2. Materials and methods

### 2.1. Animals

Breeding pairs of homozygous female and hemizygous male B6.129P2-*Fmr1*^*TM*1*Cgr*^/J mice (*Fmr1*-KO; #:003025) (The Dutch-Belgian Fragile X Consortium, 1994), and BTBR *T*^+^ *Itpr3^tf^*/J mice (BTBR; #:002282) (Petkov et al., 2004; Timmermans et al., 2017) were purchased from Jackson Laboratories, Bar Harbor, ME, USA while C57BL/6JRj (B6) breeding pairs were purchased from Janvier Labs, Le Genest-Saint-Isle, France (# SC-C57J-F). All mice were further bred at the AAALAC-accredited animal facility of QPS Austria GmbH, Grambach, Austria. All animals were housed in single-sex groups of 3−5 mice per cage and kept in ventilated cages under a 12:12 h dark-light cycle and a temperature of 21 ± 1°C at 40 to 70% humidity. Dried pelleted standard rodent chow (Altromin^®^, Lage, Germany) and water were provided *ad libitum*. All behavioral tests were performed during the light cycle phase (07:00 a.m.−4:00 p.m.). Each test was continuously performed at the same time of the day. Animals were allocated to experimental groups according to their strain and initial body weight. The use of animals was subject to the statutory provision under the Austrian Animal Welfare Act and was approved by the Styrian government, Austria (ABT13-67744/2020).

### 2.2. Experimental design

A total of 45 male BTBR and *Fmr1*-KO mice with a starting age of 4 weeks were allocated to receive either LP-211 (LP; 3 mg/kg, provided by M. Leopoldo, Bari, Italy. Purity > 98%), R-Baclofen (Bf; 1.5 mg/kg, Sigma Aldrich, St. Louis, MO, USA, G013) or vehicle (PEG400 20% v/v + Tween80 10% v/v + 0.9% saline). In addition, control groups of 15 male B6 animals received vehicle only. All animals were treated with an intraperitoneal injection 30 min before each behavioral test performed after age of 5 weeks as these strains are expected to exhibit relevant ASD behaviors mostly from this age (Leopoldo et al., 2011; Silverman et al., 2015).

**FIGURE 1:**
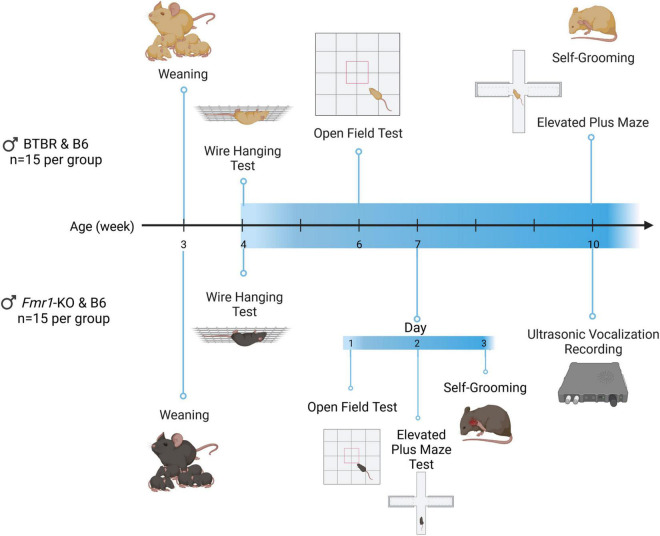
Timeline of BTBR and *Fmr1*-KO mice experiments. Animals were weaned at the end of week 3. Wire hanging, open field, grooming, and elevated plus maze test were performed at the age of 4, 6, and 10 weeks for BTBR mice. For *Fmr1*-KO mice, the wire hanging test was performed at the age of 4 weeks. The open field, grooming and elevated plus maze were performed on the 1^st^ to 3^rd^ day of week 7, respectively. Ultrasonic vocalization recording was performed at week 10. Created with BioRender.com.

Based on previous findings of these mouse models (Kazdoba et al., 2014; Silverman et al., 2015; Meyza and Blanchard, 2017; Costa et al., 2018; Zeidler et al., 2018), the behavioral test battery as well as compound dosing selection were designed as indicated in Figure 1. BTBR and *Fmr1*-KO mice are biologically and developmentally different, thus a different experimental design was applied for these animals. Each of these strains show ASD phenotypes such as modulated anxiety, at particular age stages, the testing timeline was therefore adjusted for each strain accordingly. Therefore, each analyzed parameter was scheduled based on phenotypic characteristics of BTBR and *Fmr1*-KO mice. All animals were weaned on postnatal day 21. Before each behavioral test, animals were habituated to the test room for 45 min.

Animal’s body weight was recorded every 7 days throughout the whole study. Veterinarian care was provided to animals that lost body weight by providing daily wet food pellets on the cage floor starting from day of weaning (postnatal day 21) until 6 weeks of age.

#### 2.2.1. Wire hanging test

The wire hanging test was performed to detect neuromuscular abnormalities of muscle strength within the cut-off time of 300 sec (Hashemi et al., 2007; van Putten, 2014). For the test, a wire cage lid was taped with duct tape around the perimeter to prevent the mouse from walking off the edge. The animal was then placed on the cage grid. The grid was afterward slightly shaken so the animal tightly grabs the wires. Then, the grid was turned upside down. The wire cage lid was held at a height of approximately 50−60 cm above a soft underlay. This distance was high enough to prevent the mouse from jumping down, but not high enough to cause harm in the event of a fall. The latency to fall off the grid was measured in seconds.

#### 2.2.2. Open field test

Mice were subjected to the open field test to explore locomotion and anxiety (Seibenhener and Wooten, 2015). In brief, each mouse was placed in the center of the Plexiglas open field box (48 × 48 cm) surrounded by two rows of infrared photograph beams to detect motion and rearing (standing on the hind paws) and given 30 min to freely explore the box at day light room lighting conditions. The vertical and horizontal movements of animals were recorded within 5 min intervals by a computer-operating tracking system (TSE-System^®^, Berlin, Germany). Multiple parameters such as activity, hyperactivity, moved distance, thigmotaxis, rearing number, and duration, and time spent in the center of the box were calculated automatically by the ActiMot Software (TSE-System^®^, Berlin, Germany).

Activity: Time in seconds animals were active with a speed of > 5 m/s. Hyperactivity: Time in seconds animals were active with a speed of > 15 m/s. Moved distance: total distance that was moved by the animal during the test time measured in centimeters. Thigmotaxis: relative time in seconds that the animal spent in the periphery versus the center of the arena indicating an anxiety-like behavior of the animal when spending more time in the periphery. Rearing number and duration: total number and time in seconds that animals spent standing on the hind legs to observe the environment.

#### 2.2.3. Elevated plus maze test

In addition to the open field test, general anxiety-like behavior of animals was evaluated with the elevated plus maze test (Lister, 1987). In brief, each mouse was allowed to freely explore the elevated plus maze apparatus consisting of two open arms (25 × 5 cm), two enclosed arms (25 × 5 × 16 cm), and a central zone (5 × 5 cm) for 5 min while being video recorded. The cumulative time spent and the frequency of entry in either arm or the central zone of the maze were evaluated by the Noldus EthoVision XT 8.5 (Wageningen, Netherlands) software for the whole 5 min test duration. The test was performed under red light illumination.

#### 2.2.4. Self-grooming test

Animals were scored for spontaneous self-grooming behavior (Silverman et al., 2010b; Kalueff et al., 2016). In brief, each mouse was placed in a clean cage, free of bedding, for 15 min and video recorded at day light room lighting conditions. The first 5 min were considered habituation and the following 10 min were evaluated as test session. The cumulative duration of self-grooming and number of grooming episodes were scored with Noldus Observer XT 15 (Wageningen, Netherlands) software.

#### 2.2.5. Ultrasonic vocalizations

##### 2.2.5.1. Familiarization of the male mouse with the smell of female

One week before ultrasonic vocalization (USV) recording, male mice were familiarized with female odor by placing 1 male with an estrous female mouse in a clean cage covered with fresh bedding for 5 min. Mice were observed carefully to prevent any sexual activity.

##### 2.2.5.2. Estrous cycle induction in female mice

Only urine of females in the estrous cycle were used to excite male mice. To induce estrus cycle, adult female C57BL/6JRj mice were subcutaneously injected with β-estradiol (1 μg/50 μl, E-8515, Sigma Aldrich, St. Louis, MO, USA) and progesterone (16 mg/ml, P-0130, Sigma Aldrich, St. Louis, MO, USA) in corn oil (C-8267, Sigma Aldrich, St. Louis, MO, USA) each 48 and 6 h before familiarization or performance of the actual USV recording (Edwards, 1970; Nah et al., 2011).

##### 2.2.5.3. Urine collection from estrous females

To collect urine, each female mouse was grasped, and the urine was collected on small pieces of filter paper. This filter paper was placed in the clean cage containing the male study mouse right before pressing the start button of the recording program.

##### 2.2.5.4. USV recording day

On the test day, animals were habituated in a separate soundproof room. Before recording in the same room, each mouse was placed in a cage with fresh bedding. Recordings were performed with an UltraSoundGate Condenser Microphone (CM16; Avisoft Bioacoustics, Nordbahn, Germany), connected *via* an Avisoft UltraSoundGate 416 USB Audio device (Avisoft Bioacoustics, Nordbahn, Germany) to a personal computer. Acoustic data were recorded with a sampling rate of 250,000 Hz in 16-bit format. After placing a filter paper with urine of an estrous female in the mouse cage, the recording was started under red light condition. Vocal emission was recorded for 5 min.

##### 2.2.5.5. USV data analysis

For analysis, recordings were transferred to SASLab Pro (Avisoft Bioacoustics, Nordbahn, Germany), and a fast Fourier transformation was conducted (512 FFT length, 100% frame, Hamming window and 75%-time window overlap). For automatic parameter measurement setup, the maximum changes were set to 10 pixels with 5 ms minimum duration and 10 ms hold time. The total number of calls and the latency to initiate the first call were measured and evaluated for the whole 5 min of the recordings.

### 2.3. Statistical analysis

Data analysis was performed by GraphPad Prism™ 9.2 (GraphPad Software Inc., San Diego, CA, USA). All graphs present group means with standard error of the mean (SEM). The significance level was set at *p* < 0.05. Group means were compared using One-way or Two-way analysis of variance (ANOVA), with a subsequent *post hoc*; Dunn’s Multiple Comparison or Fischer’s LSD test. For comparison of two groups, Student’s *t*-test was performed. Significances were defined as *p < 0.05; ^**^p < 0.01; ^***^p < 0.001.

Pearson’s Correlation Coefficient (r) was used to compare animals’ body weight and latency to fall off the wire in the wire suspension test. Moreover, the results of the Pearson’s Correlation test of BTBR and *Fmr1*-KO mice were compared using Fischer’s *Z* transformation (Eid et al., 2011). To this aim, the online tool https://www.psychometrica.de/correlation.html#fisher was used. *Z*- and *p*-values are provided.

The exact group size and utilized statistical tests are mentioned in the figured legends.

All raw data of this study are provided in Supplementary Table 1.

## 3. Results

### 3.1. Wire hanging test reveals motor deficits of BTBR mice starting at the early stage of life

**FIGURE 2:**
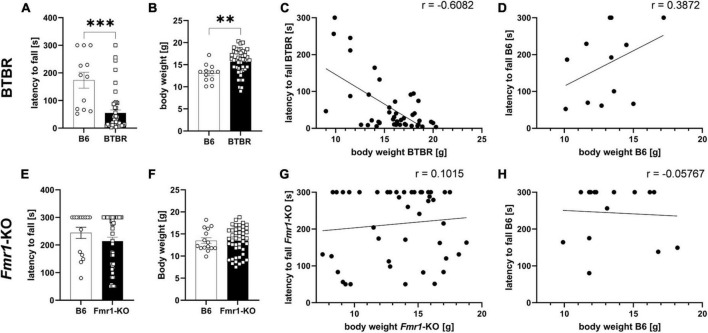
Wire hanging test. Latency to fall off the wire **(A,E)**, body weight measurements after the wire hanging test **(B,F)**, correlation curves of body weight and latency to fall off the wire **(C,D,G,H)** of BTBR **(A–D)** and *Fmr1*-KO **(E–H)** compared to B6 mice at 4 weeks of age. Cut-off time of 300 s. Unpaired *t*-test; B6: *n* = 15, BTBR/*Fmr1*-KO: *n* = 45, mean ± SEM. ^**^p < 0.01, ^***^ *p* < 0.001.

The wire hanging test was performed as part of the general health check before treatment start of the efficacy study. Based on body weight results, mice were allocated to treatment groups, so that all groups would present similar body weight averages. The wire hanging test evaluating neuromuscular changes was performed at 4 weeks of age. The body weight of all animals was measured immediately thereafter and correlated with the wire hanging results. Results show that BTBR mice fell significantly earlier off the wire compared to B6 mice (Figure 2A; *p* ≤ 0.001). Body weights of BTBR mice was significantly higher compared to B6 mice (Figure 2B; *p* = 0.004). In addition, results of the wire hanging test were correlated with body weight data (Figures 2C, D). There was a significant negative correlation of body weight and the latency to fall in BTBR mice (Figure 2C; *r* = −0.6082; *p* ≤ 0.0001) but no correlation of these parameters in B6 mice (Figure 2D; *r* = 0.3872; *p* = 0.2137). Moreover, there was a significant difference between correlations of time hanging and body weight in BTBR mice and the B6 control group (*z* = −3.028; *p* = 0.001).

The *Fmr1*-KO mice, however, did not reveal any motor dysfunction by showing a comparable latency to fall and body weight levels compared to B6 control mice (Figures 2E, F). There was no significant correlation between body weight and the latency to fall in *Fmr1*-KO (Figure 2G; *r* = 0.1015; *p* = 0.5072) and B6 (Figure 2H; *r* = −0.05767; *p* = 0.8382). There was no significant difference between correlations of latency to fall off the wire and body weight in *Fmr1*-KO mice and the B6 control group (*z* = 0.488; *p* = 0.313).

### 3.2. BTBR mice’s body weight is decreased by R-Baclofen treatment

**FIGURE 3:**
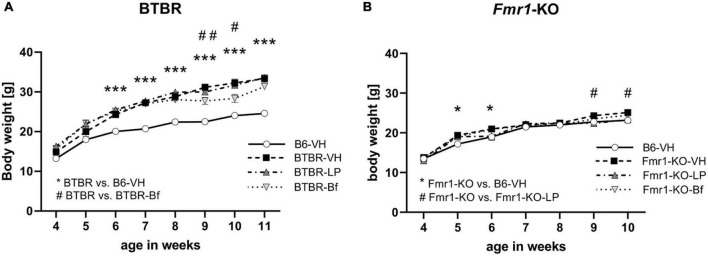
Weekly body weight measurements in BTBR **(A)** and *Fmr1*-KO **(B)** animals after treatment with LP-211, R-Baclofen, or vehicle. Two-way ANOVA followed by uncorrected Fisher’s LSD *post hoc* test. *n* = 15 per group, Mean ± SEM. All versus BTBR/*Fmr1*-KO vehicle-treated (VH) mice. ^*/#^*p* < 0.05, ^##^*p* < 0.01, ***p < 0.001.

Weekly body weight measurement of *Fmr1*-KO and BTBR mice showed that BTBR mice are heavier than B6 mice (Figure 3A; ANOVA: genotype, *p* < 0.001; age, *p* < 0.001; interaction, *p* < 0.001; *post hoc* test, *p* = 0.001). After one treatment with R-Baclofen at the age of 6 weeks and 2 treatments at the age of 10 weeks, BTBR mice lost about 10% of body weight at the age of 9 and 10 weeks (Figure 3A; *p* ≤ 0.03). Veterinarian care was provided to animals that lost body weight by providing daily wet food pellets on the cage floor until animals gained back the lost body weight. LP-211 treatment did not affect BTBR animals’ body weight.

*Fmr1*-KO mice showed a similar progression of body weight gain compared to B6 mice. Wet food pellets were provided to animals that lost body weight. *Fmr1*-KO mice showed higher body weights in week 5 and 6 than B6 mice but differences faded with age (Figure 3B; ANOVA: genotype, *p* = 0.42; age *p* < 0.001; interaction, *p* = 0.002; *post hoc* test, *p* = 0.04). In week 9 and 10, *Fmr1*-KO mice treated with LP-211 were heavier, as the vehicle-treated *Fmr1*-KO mice seem to stop gaining weight (Figure 3B; *p* = 0.04). R-Baclofen treatment did not affect the body weight of *Fmr1*-KO mice.

From this point onward, animals were treated with R-Baclofen, LP-211 or vehicle 30 min prior to each test.

### 3.3. Hyperactivity in *Fmr1*-KO and anxiety in BTBR mice are measurable in the open field test

**FIGURE 4:**
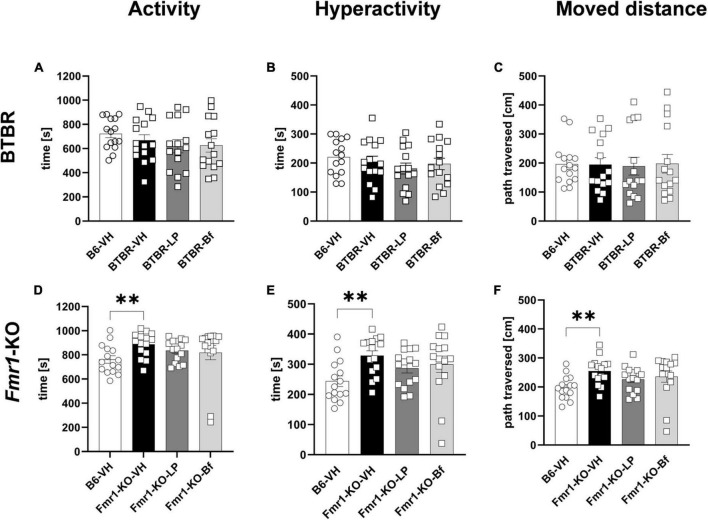
Locomotor activity of BTBR and *Fmr1*-KO mice in the open field test. Activity **(A,D)**, hyperactivity **(B,E),** and moved distance during a 30 min trial **(C,F)** of BTBR **(A–C)** and *Fmr1*-KO **(D–F)** mice. One-way ANOVA followed by uncorrected Fisher’s LSD **(D,F)** or uncorrected Dunn’s **(E)** *post hoc* test. *n* = 15 per group. Mean ± SEM. All groups versus BTBR/*Fmr1*-KO vehicle-treated (VH) mice. ^**^p < 0.01.

Locomotor activity was measured during a 30 min trial in BTBR and B6 mice at the age of 6 weeks. Hyperactivity did not show a difference between BTBR and B6 mice (Figures 4A, B). Consistent with activity and hyperactivity levels, there were no differences observed in the moved distance between BTBR and B6 mice. Acute LP-211 and R-Baclofen treatment did not affect these parameters in BTBR mice (Figures 4A-C).

*Fmr1*-KO mice at 7 weeks of age, however, displayed a significantly higher activity and hyperactivity (Figures 4D, E; *p* = 0.004; *p* = 0.003, respectively) compared to B6 mice during the 30 min open field test. *Fmr1*-KO mice moved a longer distance (Figure 4F; *p* = 0.002) than B6 mice in the open field box.

**FIGURE 5:**
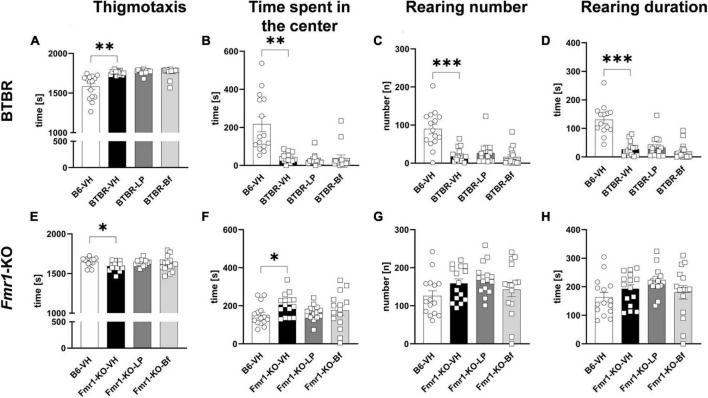
Anxiety measurement of BTBR and *Fmr1*-KO mice in the open field test. Thigmotaxis **(A,E)**, time spent in the center of the open field box **(B,F)**, rearing number **(C,G)**, and rearing duration **(D,H)** of BTBR **(A–D),** and *Fmr1*-KO **(E–H)** mice. One-way ANOVA followed by uncorrected Fisher’s LSD **(E–H)** or uncorrected Dunn’s **(A–D)** *post hoc* test. *n* = 15 per group. In graph **(D)**, one value had to be excluded from B6-VH group due to a software error. Mean ± SEM. All groups vs. BTBR/*Fmr1*-KO vehicle-treated (VH) mice. *p < 0.05, ^**^p < 0.01, ^***^ *p* < 0.001.

Evaluation of parameters related to anxiety showed a significantly higher thigmotaxis (Figure 5A; *p* = 0.001) and shorter time spent in the center of the box (Figure 5B; *p* = 0.001) in BTBR mice compared to B6 mice. Anxiety in BTBR mice was further confirmed by the observed lower rearing number and rearing duration compared to B6 mice (Figures 5C, D; *p* ≤ 0.001). Acute LP-211 and R-Baclofen did not alleviate anxiety-like behavior observed in BTBR mice compared to vehicle-treated BTBR mice (Figures 5A-D).

*Fmr1*-KO mice presented significantly less thigmotaxis compared to B6 mice (Figure 5E; *p* = 0.03). Moreover, low anxiety in *Fmr1*-KO mice was validated by the longer time animals spent in the center of the open field box compared to B6 mice (Figure 5F; *p* = 0.03). The rearing activity was comparable between *Fmr1*-KO and B6 mice. Acute treatment of LP-211 and R-Baclofen did not affect these parameters in *Fmr1*-KO mice, indicating no sedating effect of these drugs at the applied doses (Figures 5E-H).

### 3.4. *Fmr1*-KO mice display reduced anxious behavior in the elevated plus maze test

**FIGURE 6:**
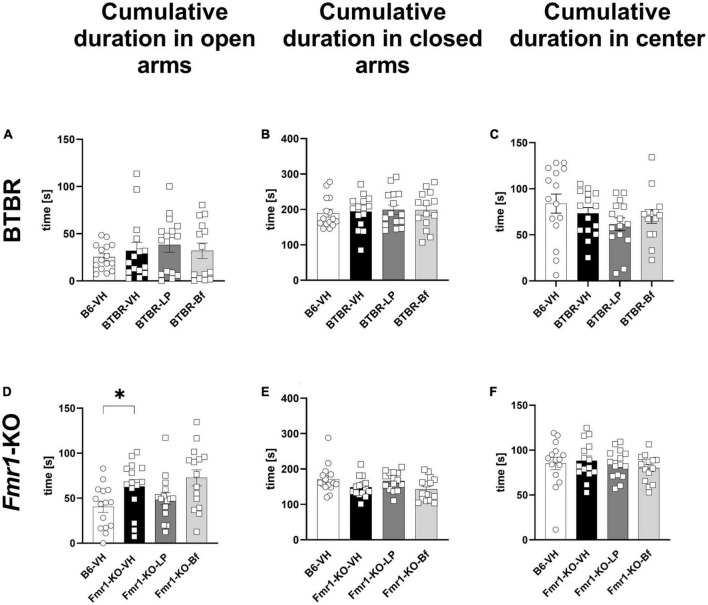
Cumulative duration in closed arms, open arms, and central zone of the elevated plus maze test of BTBR and *Fmr1*-KO mice. Total time spent in open arms **(A,D)**, closed arms **(B,E)** and central zone **(C,F)** in seconds during a 5-min session. One-way ANOVA followed by uncorrected Fisher’s LSD **(A,B**,**D–F)** or uncorrected Dunn’s **(C)** *post hoc* test. *n* = 15 per group. Mean ± SEM. All groups vs. BTBR/*Fmr1*-KO vehicle-treated (VH) mice. *p < 0.05.

Based on the observed results from the open field test, we aimed to further explore anxiety-like behavior by the widely used elevated plus maze (EPM) test in BTBR and *Fmr1*-KO mice at the age of 10 and 7 weeks, respectively. BTBR mice spent the same amount of time in open and closed arms and the center of the maze compared to B6 mice (Figures 6A–C). *Fmr1*-KO mice spent significantly more time in the open arms of the EPM compared to B6 mice (Figure 6D; *p* = 0.03). The cumulative duration in the closed arms, as well as central zone of the maze was similar between *Fmr1*-KO and B6 mice (Figures 6E, F; *p* = 0.06 and *p* = 0.68, respectively).

**FIGURE 7:**
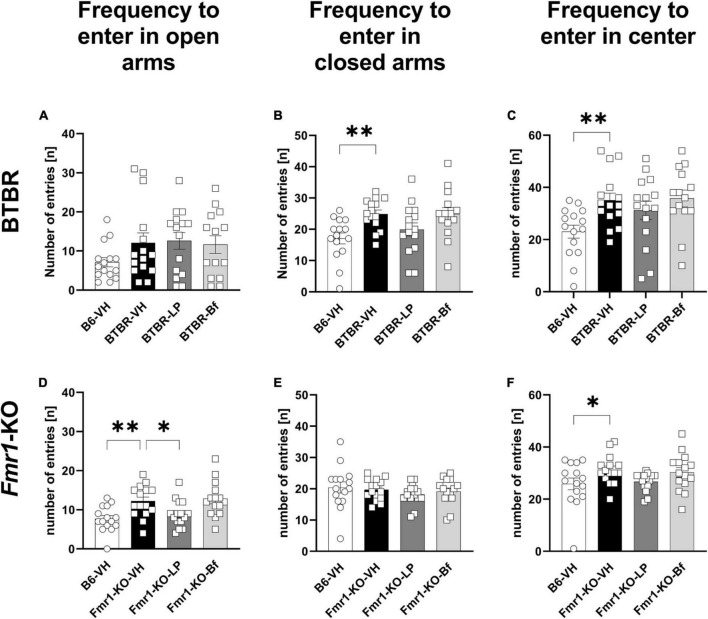
Frequency of entries to closed arms, open arms, and central zone of the elevated plus maze of BTBR and *Fmr1*-KO mice. Total number of entries to open arms **(A,D)**, closed arms **(B,E)** and central zone **(C,F)** during a 5 min session under red light. One-way ANOVA followed by uncorrected Fisher’s LSD **(B,C,F)** or uncorrected Dunn’s **(A,D,E)** *post hoc* test. *n* = 15 per group. Mean ± SEM. All groups versus BTBR/*Fmr1*-KO vehicle-treated (VH) mice. *p < 0.05, ^**^p < 0.01.

Examining the number of entries, BTBR mice entered the open arms as many times as B6 mice (Figure 7A). However, BTBR mice entered the closed arms more frequently than B6 mice (Figure 7B; *p* = 0.004). Furthermore, the BTBR mice entered the central zone of the maze more frequently than B6 mice (Figure 7C; *p* = 0.006). Acute LP-211 treatment of BTBR mice had neither significant effect on the number of entries to the closed arms nor the central zone of the EPM compared to the vehicle-treated BTBR (Figures 7B, C). There were no changes observed upon R-Baclofen injection in BTBR mice for the cumulative durations as well as frequencies to enter the open, closed, or central zone of the EPM compared to vehicle-treated BTBR mice. The frequency to enter the open arms was significantly higher in *Fmr1*-KO than B6 mice (Figure 7D; *p* = 0.003), while the frequency to enter the closed arms was similar between B6 and *Fmr1*-KO mice (Figure 7E; *p* = 0.07). None of the treatments was able to modulate this parameter in *Fmr1*-KO mice. *Fmr1*-KO mice entered the central zone of the EPM significantly more often compared to B6 mice (Figure 7F; *p* = 0.03). A decreased frequency to enter the open arms was observed in LP-211 treated *Fmr1*-KO mice compared to vehicle-treated *Fmr1*-KO mice (Figure 7D; *p* = 0.03). R-Baclofen treatment did not change the frequency of entries in open and closed arms and central zone of the EPM in *Fmr1*-KO mice compared to vehicle-treated *Fmr1*-KO mice.

### 3.5. Acute R-Baclofen and LP-211 treatment reduced grooming behavior in *Fmr1*-KO mice

**FIGURE 8:**
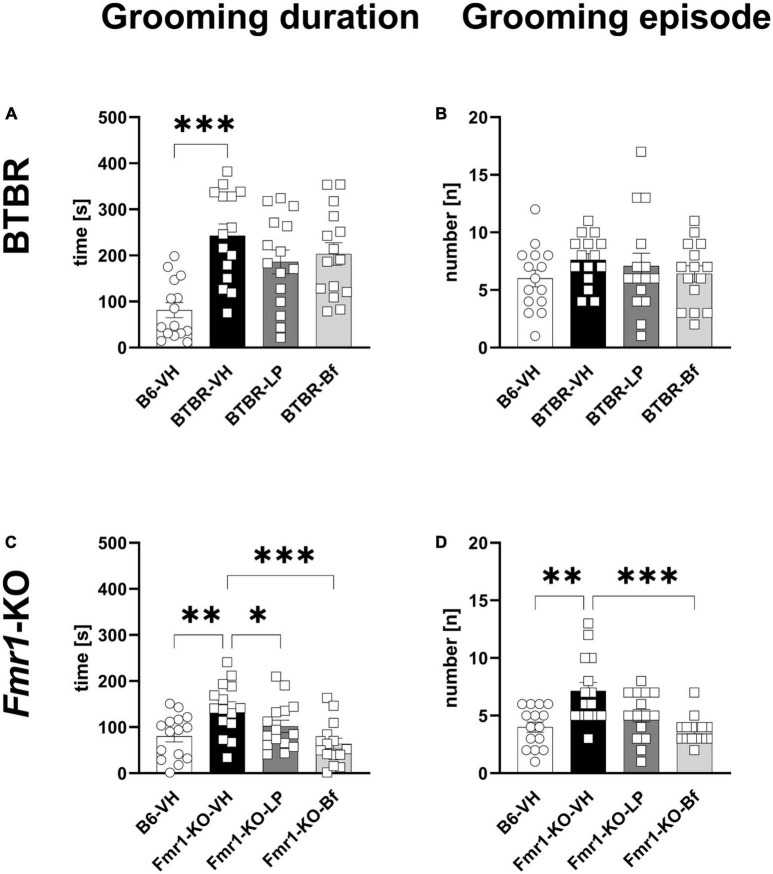
Self-grooming behavior of BTBR and *Fmr1*-KO mice. Total self-grooming duration **(A,C)** and total grooming episodes **(B,D)** during the 10 min recording session in BTBR **(A,B)** and *Fmr1*-KO **(C,D)** mice. One-way ANOVA followed by uncorrected Fisher’s LSD **(C)** or uncorrected Dunn’s **(A–D)** *post hoc* test. *n* = 15 per group. Mean ± SEM. All groups versus BTBR/*Fmr1*-KO vehicle-treated (VH) mice. *p < 0.05, ^**^p < 0.01, ^***^ *p* < 0.001.

Repetitive behavior was monitored by evaluating self-grooming in a 10 min session in BTBR and *Fmr1*-KO mice at the age of 10 and 7 weeks, respectively. BTBR mice spent significantly more time self-grooming compared to B6 mice (Figure 8A; *p* ≤ 0.001); however, there was no significant difference observed in the number of grooming episodes (Figure 8B). None of the compounds was able to reverse these parameters in BTBR mice.

*Fmr1*-KO mice spent a significantly longer time grooming with a higher number of grooming episodes during the 10 min of recording (Figures 8C, D; *p* = 0.002, *p* = 0.002, respectively). Acute R-Baclofen treatment significantly ameliorated the grooming duration as well as number of grooming episodes in *Fmr1*-KO mice (Figures 8C, D; *p* ≤ 0.001; *p* ≤ 0.001, respectively). Acute LP-211 treatment significantly reduced the grooming duration in *Fmr1*-KO mice (Figure 8C; *p* = 0.04).

### 3.6. Impaired ultrasonic vocalization emission in *Fmr1*-KO mice did not improve by R-Baclofen or LP-211 treatment

Since R-Baclofen and LP-211 treatment only alleviated repetitive behavior in *Fmr1*-KO mice, we further studied the effect of these compounds on the social interest and communication test by measuring ultrasonic vocalization in these mice. This test was chosen to measure social communication as results of this test are less variable and more reliable compared to other tests such as the automated three-chamber social interaction test as less interventions to the test animal are needed to perform the test.

**FIGURE 9:**
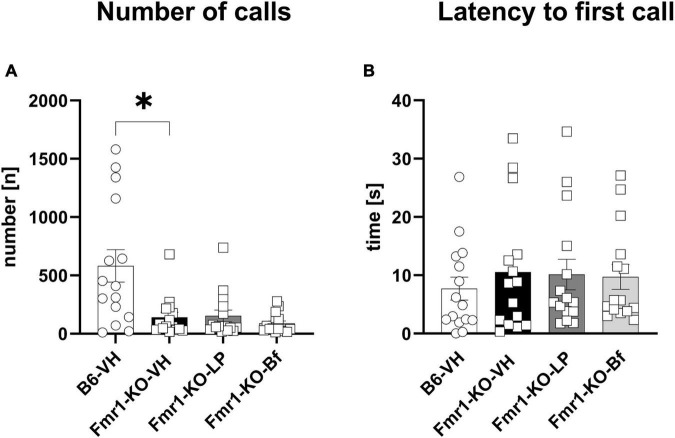
Ultrasonic vocalization emission recordings while exposed to fresh urine of an estrous female. The number of emitted calls **(A)** and latency to initiate the first call **(B)** were measured. One-way ANOVA followed by uncorrected Dunn’s *post hoc* test. *n* = 15 per group. Mean ± SEM. All groups versus *Fmr1*-KO vehicle-treated (VH) mice. *p < 0.05.

Male *Fmr1*-KO mice were exposed to fresh urine of estrous females and USV calls were recorded for 5 min at the age of 10 weeks. A significantly lower number of calls was observed in *Fmr1*-KO mice regardless of treatment compared to B6 mice (Figure 9A; *p* = 0.01). The latency to initiate the first call did not differ between *Fmr1*-KO and B6 mice and did not depend on the treatment (Figure 9B). R-Baclofen and LP-211 treatment did not affect these parameters in *Fmr1*-KO mice compared to vehicle-treated *Fmr1*-KO mice.

Overall, the results of the behavioral characterization and the efficacy study showed that male BTBR mice at the age of 4−10 weeks are a suitable model to study ASD symptoms, such as motor deficits, repetitive behavior, anxiety, and stress-like behavior. Acute R-Baclofen and LP-211 treatment could not ameliorate these alterations in BTBR mice. *Fmr1*-KO mice presented ASD-like behavior in terms of hyperactivity, repetitive behavior as well as communication impairments at the age of 4−10 weeks. Acute R-Baclofen and LP-211 treatment ameliorated repetitive behavior in *Fmr1*-KO mice.

## 4. Discussion

In the present study, we behaviorally characterized two different ASD mouse models, BTBR and *Fmr1*-KO, and evaluated the efficacy of the GABA_B_ receptor agonist R-Baclofen and the serotonin 5-HT_7_ receptor agonist LP-211 in these models. Animals were tested for locomotor activity, anxiety, stereotypic, and repetitive behaviors. In addition, the sociability of *Fmr1*-KO mice was evaluated within the treatment efficacy part of the study.

### 4.1. Behavioral characterization of BTBR mice

Motor dysfunction in BTBR mice has been previously shown by Xiao et al. (2020) evaluating it from 2 weeks to 5 months of age. Researchers showed that abnormal cerebellar development could be an important factor related to motor dysfunction as well as dystonia-like behavior in these animals. Here we report motor abnormalities in BTBR mice at the age of 4 weeks as analyzed in the wire hanging test, that negatively correlated with body weights, suggesting that the body weight has a strong impact on motor abilities: only 4 out of 45 BTBR mice could hold the wire for more than 200 s. We thus conclude that motor disability in BTBR mice starts at an early age.

Approximately half of the children with ASD meet the diagnostic criteria of hyperactivity as well as attention-deficit disorder (Murray, 2010), thus evaluating locomotor activity in ASD animal models is an important parameter to evaluate their translatability. Here, when measuring the locomotor activity in the non-social context of the open field test, 6 weeks old BTBR mice presented no differences in activity and hyperactivity. A previous report of the same test performed with 22 days old BTBR mice showed that they traveled more than B6 controls in the first 15 min of the test session that was performed under red light (McFarlane et al., 2008). Furthermore, another study showed that 8 weeks old BTBR mice present hyperactivity compared to wild type control mice by an increased distance moved in all zones in the open field test (Xiao et al., 2020).

However, another study measured the distance moved in the open field test in 15 months old male BTBR mice for 30 min under room lightening, in which BTBR mice moved a comparable distance than B6 controls (Jasien et al., 2014). Differences in these results can be explained by different test settings like the age of animals, duration of recording, and room light condition. Overall, BTBR mice appear to present hyperactivity behavior at a young age until early adolescence but lose this phenotype during adulthood.

We further evaluated the anxiety level of BTBR mice by measuring anxiety parameters in the open field and EPM at the age of 6 and 10 weeks, respectively. BTBR mice presented a high anxiety profile both in open field and EPM test. When considering the total amount of the time spent in the open field box, which is a sum of thigmotaxis and time spent in the center, BTBR mice strongly exhibited anxious behavior by spending most of the time at the outer boundaries of the box and the least time in the center. Previous reports showed an anxiogenic profile of 14−17 weeks old BTBR mice by using the EPM as animals showed a significantly lower number of entries in the open arms. Authors mention that the lightening of the test room was a stressor to which BTBR mice show a strong response, manifesting high stress-like behavior in BTBR mice (Pobbe et al., 2011). Moreover, it is worth to mention that BTBR mice are shown to have impaired photoreceptor function and therefore present atypical visual perception which mimics some subset of ASD patients (Cheng et al., 2020). Another research group reported anxiety in 8−12 months old BTBR mice compared to wild type mice by the elevated open platform test, where BTBR mice spent more time at the edges and less time in the center of the open platform (Chao et al., 2018). Another study assessing anxiety-like behavior in aged (19−21 months old) BTBR mice showed a decreased anxiety-like behavior compared to existing results in younger mice by showing that animals spent less time in the closed arms of the EPM (O’Connor et al., 2021). We thus conclude that BTBR mice exhibit anxiety and stress-like behavior at adolescent age.

Furthermore, we confirmed repetitive behavior in the self-grooming test, as 10 weeks old BTBR mice spent a longer time self-grooming compared to controls. These results are consistent with previous reports testing BTBR mice for self-grooming from postnatal day 18 to 60 (McFarlane et al., 2008) as well as weeks 7 to 8 (Amodeo et al., 2014). Furthermore, O’Connor et al. (2021) showed higher repetitive behavior of self-grooming in 19−21 months old BTBR mice compared to wild type mice. We thus conclude that BTBR mice exhibit repetitive behavior at adolescent age, and, based on previous reports, maintain this behavior lifelong.

### 4.2. Behavioral characterization of *Fmr1*-KO mice

Based on a study by Pietropaolo et al. (2011), the B6 background is more suitable to study core autism symptoms of social impairments, repetitive behaviors, as well as hyperactivity compared to the FVB background. Here, *Fmr1*-KO mice on a B6 background were thus used to evaluate ASD-like behaviors. The results obtained from the behavioral characterization of *Fmr1*-KO mice are consistent with previous reports (Michalon et al., 2012; Kramvis et al., 2013; Hébert et al., 2014; Kazdoba et al., 2014; Gantois et al., 2017; Gaudissard et al., 2017). In summary, *Fmr1*-KO mice did not show any motor dysfunction and presented comparable body weights as B6 mice. Although a micro-computerized tomography analysis performed by Leboucher et al. (2019) showed that the deficiency in the *Fmr1* gene impacts skeleton and bone morphology in 16 weeks old *Fmr1*-KO mice, it does not influence the animal’s body weight and motor performance. Moreover, in *Fmr1*-KO mice the body weight does not correlate with the latency to fall off the wire in the wire suspension test. Thus, a heavier mouse does not necessarily fall off earlier compared to a lighter mouse.

The observed differences in the latency to fall off the wire in control animals of BTBR and *Fmr1*-KO studies could be caused by the different breeding pairs that were used for each study.

Results of the behavioral characterization of *Fmr1*-KO mice in the open field and EPM test indicate hyperactivity as well as lower anxiety in this mouse model consistent with previous reports performed in 2−5 months old *Fmr1*-KO mice using the same tests (Mineur et al., 2002; Qin et al., 2002; Saré et al., 2016). Studies evaluating overlapping anxiety and hyperactivity in humans show that around 50% of adults and up to 30% of children with attention deficit hyperactivity disorder (ADHS) meet both diagnostic criteria (Pliszka, 2000; Michelini et al., 2015; Mulraney et al., 2018; van der Meer et al., 2018). Though it must be pointed out that the complexity of neuropsychiatric disorder symptoms such as hyperactivity such as excessive activity, decreased sleep need, disturbed behavior, etc., challenges the interpretation of data from animal studies (Nestler and Hyman, 2010; Yen et al., 2013). Hence, the lower anxiety-like behavior observed in *Fmr1*-KO mice detected by a higher frequency to enter and more time spent in the open arms of the EPM test could be affected by the higher activity and hyperactivity observed in these mice. Although hyperactivity might influence the results of anxiety analyses, evaluation of *Fmr1*-KO mice in the EPM and open field test definitively demonstrates that these animals have a reduced anxiety.

Repetitive behaviors are considered a key characteristic of ASD (Caldwell-Harris, 2021), thus reproducing them in animal models is crucial for the use of these models for further ASD research. *Fmr1*-KO mice showed repetitive behaviors by spending more time as well as more episodes of self-grooming compared to controls. These data are consistent with previous reports about the grooming behavior of 3 months old *Fmr1*-KO mice on a B6 background (Mines et al., 2010; Pietropaolo et al., 2011).

Results from the ultrasonic vocalization recording of 10 weeks old *Fmr1*-KO mice revealed impaired sociability and communication deficits in this mouse model. Some studies showed a similar average quantity of calls from adult *Fmr1*-KO compared to wild type mice. One example is the study where *Fmr1*-KO mice were bred on an FVB background and USV was recorded while exposing mice to fresh urine collected from estrus female mice for 5 min (Hodges et al., 2017). Moreover, another study reports *Fmr1*-KO on B6 background at the age of 2−3 months while directly exposed to receptive female mice for 2 min (Belagodu et al., 2016). Another study reported a reduced rate of ultrasonic calls during mating for 10 min by 2−3 months old *Fmr1*-KO mice on an FVB background compared to wild type mice and thus on a different background than the one used here (Rotschafer et al., 2012). We can conclude that not only age but also the genetic background as well as the test setting, e. g., female mice vs. urine of female mice (Hoffmann et al., 2009) as well as exposure time can affect the outcome of the ultrasonic vocalization test of adult male *Fmr1*-KO mice (Premoli et al., 2021).

### 4.3. R-Baclofen treatment

R-Baclofen, a GABA_B_ receptor agonist, has been already used in various clinical and preclinical studies to ameliorate ASD phenotypes (Cryan et al., 2004; Erickson et al., 2014; Qin et al., 2015; Berry-Kravis et al., 2017; Veenstra-VanderWeele et al., 2017; Stoppel et al., 2018; Möhrle et al., 2021). The applied drug concentration is critical, especially in studies measuring behavioral changes where deleterious side effects such as sedation or hyperactivity need to be avoided. A study showed a paradoxical effect of chronic R-Baclofen not only in the *Fmr1*-KO mouse model but also in B6 control mice by increasing activity (Zeidler et al., 2018). Another study reported the sedative effects of R-Baclofen in higher doses such as 3−5 mg/kg intraperitoneal treatment in the BTBR and C58 ASD mouse models (Silverman et al., 2015). Therefore, in the current study, the concentration of 1.5 mg/kg was carefully selected to induce disease ameliorating effects while preventing any side effects. Concentrations less than 3 mg/kg have been reported to be effectless on locomotor activity, repetitive behavior, as well as sociability in B6 mice (Silverman et al., 2015), therefore, no B6 mice were treated with R-Baclofen in the current study.

In 2005, Silverman et al. (2015) evaluated the efficacy of R-Baclofen on both sexes of BTBR mice at the age of 10−12 weeks with a single dose of 1 and 3 mg/kg 60 min prior to testing. Both doses improved sociability in BTBR mice but only the 3 mg/kg dose could improve the repetitive behavior of these mice. The same research group showed in an earlier study that 1 mg/kg of R-Baclofen did not reverse the social impairment of male-female interaction in BTBR mice (Silverman et al., 2015). In the present study, BTBR vehicle-treated mice showed higher anxiety and higher repetitive grooming behavior. R-Baclofen treatment did not improve these parameters. Thus, it might be possible that each behavioral assay requires a different optimal dose of R-Baclofen to be effective. Chronic treatment with R-Baclofen might be more useful, not only because ASD is a lifelong disorder and would require constant treatment, but it would also blunt the observed side effects when using higher concentrations. As discussed by Silverman et al. (2015), comprehensive studies conducting both acute and chronic treatments for each behavioral assay would be the most effective way of assessing compound efficacy.

In *Fmr1*-KO mice, there were no improvements in hyperactivity, anxiety level, and communication detected after R-Baclofen treatment. However, acute R-Baclofen injection could significantly reduce the repetitive grooming behavior in *Fmr1*-KO mice. While both BTBR and *Fmr1*-KO are used in ASD research, (Silverman et al., 2015) they are genetically and behaviorally very different (Nestler and Hyman, 2010; Meyza and Blanchard, 2017; Nikvarz et al., 2017; Niu et al., 2017). Therefore, the different effects of R-Baclofen on BTBR and *Fmr1*-KO mice’ behavior are not unexpected. Nonetheless, as previously mentioned, a more efficient way to study compound effects, is to study it separately for each behavioral assay considering both acute and chronic administrations.

### 4.4. LP-211 treatment

The serotonin 5-HT_7_ receptor agonist LP-211 has already been used to alleviate object recognition memory and stereotypic behavior by an acute administration of 3 mg/kg in 3–4 months old *Fmr1*-KO mice but did not affect wild type mice (Costa et al., 2018). In addition, Costa et al. (2015, 2018) showed that activation of the 5-HT_7_ receptor by LP-211 reversed mGluR-LTD in the hippocampal region of *Fmr1*-KO mice and prevented internalization of AMPA receptors by stimulating the adenylate cyclase and protein kinase A. LP-211 not only reversed mGluR-LTD in *Fmr1*-KO mice but also in wild type animals, exhibiting the potential of this compound to modulate synaptic plasticity impairments in *Fmr1*-KO mice (Costa et al., 2015). In 2020, Armstrong et al. (2020) showed that improvement of pharmacological properties and pharmacokinetics of a serotonergic compound, a combination of 5-HT_1_A, 5-HT_2_C, and 5-HT_7_ receptor agonists, can ameliorate social deficits, prevent audiogenic seizures, and decrease repetitive behavior in male and female *Fmr1*-KO mice on a FVB background. Chronic treatment with a low dose of LP-211 was further shown to improve Rett-syndrome-related phenotypes such as memory deficits and anxiety (De Filippis et al., 2014, 2015). Since LP-211 was already shown to penetrate the brain within 30 min after intraperitoneal injection (Hedlund et al., 2010; Costa et al., 2018), we used acute injections of 3 mg/kg of LP-211 30 min before each testing in *Fmr1*-KO mice resulting in significantly reduced anxious behavior in *Fmr1*-KO mice.

We were able to reproduce the positive effect of an acute LP-211 administration on repetitive behavior in *Fmr1*-KO mice even at a younger age than previously used (Costa et al., 2018). Additionally, Lacivita et al. (2020) synthesized and evaluated a variety of long-chain arylpiperazine compounds with biased selectivity to 5-HT_7_R that showed higher drug-like properties and a high affinity to distinctive 5-HT_7_ receptors resulting in a significantly improved repetitive behavior in 12–16 weeks old *Fmr1*-KO mice.

Altogether, previous reports conclude that LP-211 corrects synaptic plasticity, abnormal intracellular signaling, as well as memory and repetitive behavior. From our current report, we can further conclude that LP-211 rescues some of the behavioral abnormalities including repetitive behaviors in *Fmr1*-KO mice and it seems that LP-211 can also be used to ameliorate anxiety (Leopoldo et al., 2011; Costa et al., 2015, 2018; Lacivita et al., 2017).

We did not detect a positive effect of LP-211 on behavioral abnormalities in BTBR mice. Although, previous reports showed serotoninergic axon density reduction in the hippocampus (Guo and Commons, 2017) and a rescue of repetitive behavior in BTBR mice after acute treatment with a 5-HT_6_ receptor blocker (Amodeo et al., 2021). Finally, we can conclude that repetitive behavior seemed to be linked to the serotoninergic system as reported previously and that serotoninergic compounds could improve this behavior in ASD mouse models such as *Fmr1*-KO mice (Lewis and Kim, 2009; Muller et al., 2016). Further research is needed to improve the pharmacological properties and study the effect of serotoninergic compounds such as LP-211 in other ASD mouse models.

So far, no FDA-approved compound exists to improve core symptoms of ASD. Animal studies are critical and valuable tools to investigate the treatment effects of already existing pharmacological compounds and to develop new drug candidates. Our preclinical data from the characterization of BTBR and *Fmr1*-KO mice by using a similar but strain-specific behavioral test battery supports the use of these mouse models for ASD research. Moreover, evaluating the efficacy of two existing pharmacological tools showed the value of these strains for future novel therapeutics. We could show some of the core and secondary symptoms in these models, and also point out how precisely mouse models can mimic the ASD phenotypic spectrum with a wide diversity in abnormality severity of abnormality. We observed strong hyperactivity in *Fmr1*-KO mice, but a moderate level in BTBR mice. Measurements of anxiety-like behavior revealed high levels of this behavior in BTBR mice, but lower anxious behavior in *Fmr1*-KO mice which could be explained as a side effect of the observed hyperactivity. Repetitive behavior was detected in both models. In addition, *Fmr1*-KO mice showed impaired communication confirmed by ultrasonic vocalization measurements.

Altogether these observations support the comorbidity of ASD core symptoms with motor dysfunction (Bougeard et al., 2021; Posserud et al., 2021), hyperactivity, and anxiety in ASD individuals (Avni et al., 2018; Nimmo-Smith et al., 2020). Our data support the idea that the GABAergic and serotoninergic systems play an important role for ASD phenotypic symptoms. We could improve the repetitive behavior, one of the core symptoms of ASD, observed in *Fmr1*-KO mice with only a single injection of a low dose of R-Baclofen or LP-211.

Although ASD symptoms of BTBR and *Fmr1*-KO mice and the impact of R-Baclofen and LP-211 have been studied previously, only a fraction of the age-related ASD phenotype of these models are understood so far. Moreover, as observed in the current study, each compound mostly shows a slight effect on the behavioral abnormalities of BTBR and *Fmr1*-KO mice. A combined treatment of R-Baclofen and LP-211 should be considered as a strategy for future ASD studies.

## Data availability statement

The original contributions presented in this study are included in the article/Supplementary material, further inquiries can be directed to the corresponding author.

## Ethics statement

This animal study was reviewed and approved by Amt der Steiermärkischen Landesregierung, Abteilung 13 – Umwelt und Raumordnung Austria.

## Author contributions

SS designed, performed, analyzed all the experiments, prepared, and corrected the manuscript. SF performed the main revision and corrected the manuscript. MD supervised SS and contributed to the experimental design. RR interpreted the results. ML and EL provided the LP-211 compound and reviewed the manuscript. BC supervised SS and reviewed the manuscript. BH-P and MP conceived the study, interpreted the data, and edited the manuscript. All authors contributed to the article and approved the submitted version.
